# Templating chiral silver assemblies in three dimensions

**DOI:** 10.1038/s42004-021-00546-7

**Published:** 2021-07-21

**Authors:** Andrew J. Bissette

**Affiliations:** Communications Chemistry, https://www.nature.com/commschem/

## Abstract

DNA-templated synthesis of chiral inorganic assemblies often requires chemical modification of the template. Now, a route exploiting the native chemistry of unmodified DNA origami templates provides access to 3D chiral assemblies.

Templated synthesis using DNA origami holds great potential to precisely control the size and shape of chiral inorganic nanoparticle assemblies, but typically require chemical modification of the DNA to achieve site selectivity. Now, an international collaboration led by Huajie Liu from Tongji University, China, reports a method for the construction of 3D assemblies of silver nanoparticles that exploits the intrinsic reactivity of nucleobases (10.1021/jacs.1c00363)^1^.

Methods for preparing chiral nanoparticle assemblies often involve inclusion of moieties, such as thiols, capable of immobilising nanoparticles at desired positions. Liu and coworkers previously^2^ reported that 2D DNA origami can strongly coordinate low-valent metal ions such as Cu(II) and Ag(I) at particular regions of the polymer, called protruding clustered regions. These pcDNA sites can be engineered from three single-stranded DNA sequences, allowing the design of site-specific nucleation sites. But this method required a solid support, limiting its application to the preparation of 2D assemblies at the surface.

To overcome this, the team exploited the interaction of diamine Ag(I) complexes with the natural nucleobases of single-stranded DNA. “We found that single-stranded DNA protrusions without chemical modification could direct the nucleation and growth of silver precursors under a mild reductive environment,” explains Liu. They designed a DNA origami scaffold bearing pendant single-stranded pcDNA sites, which are arranged in a helical pattern around a double-standed core. Sequential coordination and chemical reduction of the silver allowed controlled metalation in the desired chiral configuration. Simply by altering the design of the pcDNA sites in the origami template, inorganic helices of controlled helical screw sense and pitch could be prepared.

The mechanism of chelation is less straightforward than might be anticipated, imparting site selectivity to the method. Density functional theory calculations suggest a synergistic combination of interactions are at play, including covalent coordination, hydrogen bonding, and ion-π interactions, all of which serve to induce nucleation and growth of silver particles at pcDNA sites (Fig. 1).

**Fig. 1:**
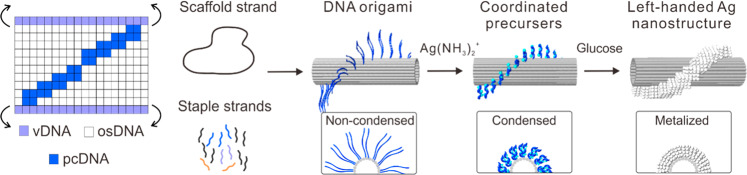
Site-selective nucleation of silver nanoparticles on native DNA. Incorporation of pcDNA sites into a DNA origami template allows for site-selective nucleation of silver-amine complexes in solution, yielding 3D chiral inorganic structures after reduction with glucose. Reprinted with permission from Zhang et al.^1^. Copyright 2021 American Chemical Society.

The approach is by no means limited to the preparation of helical structures. “Varying the chiral structures is easily done by changing the shape of the template,” says Liu. Deposition of patterns on either face of rectangular DNA origami templates offers a route to bifacial assemblies with unconventional configurations, for example. A full exploration of the many variables influencing the spectral properties, which vary with configuration, remains for future work.
